# A Digital Atlas to Characterize the Mouse Brain Transcriptome

**DOI:** 10.1371/journal.pcbi.0010041

**Published:** 2005-09-23

**Authors:** James P Carson, Tao Ju, Hui-Chen Lu, Christina Thaller, Mei Xu, Sarah L Pallas, Michael C Crair, Joe Warren, Wah Chiu, Gregor Eichele

**Affiliations:** 1 Program in Structural and Computational Biology and Molecular Biophysics, National Center for Macromolecular Imaging, Baylor College of Medicine, Houston, Texas, United States of America; 2 Verna and Marrs McLean Department of Biochemistry and Molecular Biology, Baylor College of Medicine, Houston, Texas, United States of America; 3 Department of Computer Science, Rice University, Houston, Texas, United States of America; 4 Division of Neuroscience and Program in Developmental Biology, Baylor College of Medicine, Houston, Texas, United States of America; 5 Department of Biology, Georgia State University, Atlanta, Georgia, United States of America; 6 Max Planck Institute of Experimental Endocrinology, Hanover, Germany; Lawrence Berkeley National Laboratory, United States of America

## Abstract

Massive amounts of data are being generated in an effort to represent for the brain the expression of all genes at cellular resolution. Critical to exploiting this effort is the ability to place these data into a common frame of reference. Here we have developed a computational method for annotating gene expression patterns in the context of a digital atlas to facilitate custom user queries and comparisons of this type of data. This procedure has been applied to 200 genes in the postnatal mouse brain. As an illustration of utility, we identify candidate genes that may be related to Parkinson disease by using the expression of a dopamine transporter in the substantia nigra as a search query pattern. In addition, we discover that transcription factor *Rorb* is down-regulated in the *barrelless* mutant relative to control mice by quantitative comparison of expression patterns in layer IV somatosensory cortex. The semi-automated annotation method developed here is applicable to a broad spectrum of complex tissues and data modalities.

## Synopsis

The mammalian brain is a complex organ with hundreds of functional parts. Describing when and where genes are expressed in the brain is thus a potentially powerful method for understanding the function of gene products. In recent years, several mammalian genomes including those of human and mouse have been characterized. There are now efforts around the world that aim to determine the expression patterns for all genes in the mouse brain. To search these expression data readily, they must be placed into an atlas. The authors propose a new method for bringing such genetic data into a common spatial framework so that one can perform spatial searches and comparisons of gene expression patterns. To create this atlas, the authors developed a series of maps of the brain using a graphical modeling method called subdivision. These maps were deformed to match the shape of tissue sections, and genetic activity information was associated with the appropriate coordinates on the map. After placing 200 genes into the context of this atlas, the authors illustrate its application in discovering genes potentially involved in diseases and brain development.

## Introduction

High-resolution maps of gene expression provide important information about how genes regulate biological processes at cellular and molecular levels. Therefore, a multitude of efforts are in progress to depict gene expression at single cell resolution in specimens ranging from organs to embryos (http://mamep.molgen.mpg.de [1]; http://genepaint.org/ [2]; http://brainatlas.org/ [3]; http://mahoney.chip.org/mahoney/ [4]; http://www.ncbi.nlm.nih.gov/projects/gensat/ [5]). Common to these genome-scale projects is that they generate vast numbers of images of expression patterns that reveal the presence of transcripts or proteins in a particular cell or group of cells within a natural context. However, large collections of images are of limited usefulness per se without efficient means to mine these images and to characterize and compare gene or protein expression patterns. In analogy to the requirements for mining genomic sequence information, meaningful retrieval of expression patterns requires suitable annotation. By annotation, we mean associating sites and strengths of expression with a digital representation of the anatomy of a specimen.

The annotation approach taken by the Gene Expression Database [6] is to hand-curate published gene expression patterns using an extensive dictionary of anatomical terms. This annotation is facilitated by the Edinburgh Mouse Atlas Project (EMAP), which provides anatomical ontology relationships using a hierarchical tree [7]. Visualization is achieved by associating these terms with locations in a volumetric model [8]. The Edinburgh Mouse Atlas Project also provides tools to map in situ hybridization (ISH) images directly into a three-dimensional (3D) atlas [7]. Although hand curation is an effective method for annotation, it is not an efficient means for handling the large-scale datasets systematically collected by robotic ISH [9]. In addition, if future changes are made to anatomical designations, updating the annotation may require a laborious review of previously annotated data.

Here we present a completely novel approach that uses a geometric modeling technique to create a digital atlas of the postnatal day 7 (P7) mouse brain. This deformable atlas can then be adjusted to match the major anatomical structures present in P7 mouse brain tissue sections, accurately define the boundaries between structures, and provide a smooth multi-resolution coordinate representation of small structures. When combining this technique with a method for detecting strength of gene expression, one can efficiently and automatically annotate a large number of gene expression patterns in a way that subsequently allows queries and comparisons of expression patterns in user-defined regions of interest.

P7 mouse brain was selected as the specimen because at this developmental stage, many complex brain functions begin to be established yet the existing information on underlying molecular mechanisms is still relatively limited. We describe here the creation of a prototype 200-gene dataset generated using robotic ISH, and the application of our deformable atlas-based annotation method to this dataset. We then demonstrate the utility of the approach with two examples: searching for genes expressed in the substantia nigra, and identifying genes potentially involved in functional regionalization of the cortex.

## Results

### Construction of an Atlas Using a Subdivision Mesh Technique

**Figure 1:**
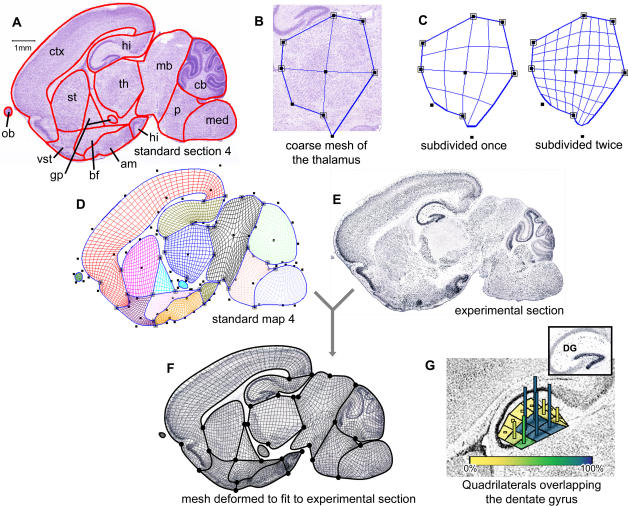
P7 Mouse Brain Atlas Construction and Application (A) Standard Nissl-stained P7 sagittal standard section number 4 with major anatomical boundaries drawn in red: amygdala (am), basal forebrain (bf), cerebellum (cb), cortex (ctx), globus pallidus (gp), hippocampus (hi), medulla (med), midbrain (mb), olfactory bulb (ob), pons (p), striatum (st), thalamus (th), and ventral striatum (vst). (B) The coarse mesh, shown here for the thalamus, is constructed by defining vertices of quadrilaterals. (C) Iterative application of subdivision generates smooth boundary curves and a smooth internal representation of smaller quadrilaterals. Fixed vertices (large squares) allow crease angles to be added to the otherwise smooth boundary curve. (D) The atlas for standard section number 4. Each coarse quadrilateral is associated with a particular anatomical structure, an association inherited during subdivision. (E) Expression pattern of *Cannabinoid receptor 1* in a section similar to standard map 4. (F) The atlas (D) is deformed by moving vertices so that the anatomical boundaries match those in the *Cannabinoid receptor 1* section (E). (G) Quadrilaterals overlying the DG (insert) were marked in 59 fitted maps using a mesh generated after two rounds of subdivision. In every section, the same four quadrilaterals were found to overlap the bulk of the DG.

In building the atlas of the P7 mouse brain, we first selected a set of 11 cresyl-violet-stained standard sagittal brain sections that approximate the 11 sagittal sections in Valverde's atlas of the postnatal mouse brain [10]. These standard sections exhibit the hemi-brain by spanning from lateral (section 1) to paramedial (section 11). The boundaries of 15 major brain structures (amygdala, basal forebrain, cerebellum, cortex, globus pallidus, hippocampus, hypothalamus, medulla, midbrain, olfactory bulb, pons, septum, striatum, thalamus, and ventral striatum) were then delineated on each of the 11 standard sections. (The boundaries for standard section 4 are shown in Figure 1A.) For each of these major structures in a standard section, we created a representation using a coarse quadrilateral mesh. Figure 1B shows an example of creating a coarse mesh for the thalamus in standard section 4. The subdivision algorithm applies an iterative refinement of this coarse mesh, resulting in a fine mesh that both smoothly overlays internal regions of structures and explicitly defines their boundaries (Figure 1C). The complete mesh across an entire section is an accurate map representing all major anatomical structures (Figure 1D). Performing the described process for all 11 standard sections resulted in 11 maps, which together constitute an atlas of the P7 mouse brain (Figure S1).

Each of the 11 maps is deformable and hence can be precisely fitted to an anatomically similar experimental sagittal brain section (e.g., Figure 1E). The shape of the fine mesh is controlled by repositioning the vertices of the coarse mesh (indicated by black squares in Figure 1B–1D). An automated global fit of the map can be used for an initial approximation of the related map to the experimental section [11]. A manual adjustment by dragging vertices into new positions then allows the map to fit the boundaries of the anatomical structures in the experimental section accurately (Figure 1F).

Anatomical substructures in the mouse brain maintain a consistent spatial relationship with neighboring structures when specimen age and strain do not change. Thus, the location of any given substructure should be consistently represented by a set of quadrilaterals in the fitted map. This important property was examined by fitting standard map 6 to 59 different experimental sections and then determining which quadrilaterals contained the dentate gyrus (DG), a substructure of the hippocampus. Although the shape of the DG and its relative position within the hippocampus varied to some extent (e.g., because of tissue compression/stretching in the sectioning process), the same four quadrilaterals always contained most of the DG, with adjacent quadrilaterals sometimes containing the edge of the DG (Figure 1G). This suggests that the subdivision mesh-based atlas not only explicitly delineates the boundaries between major structures, but can also be used to define the location of internal substructures such as the DG.

### Establishment of Annotated Gene Expression Patterns

#### Nonradioactive ISH data.

**Figure 2:**
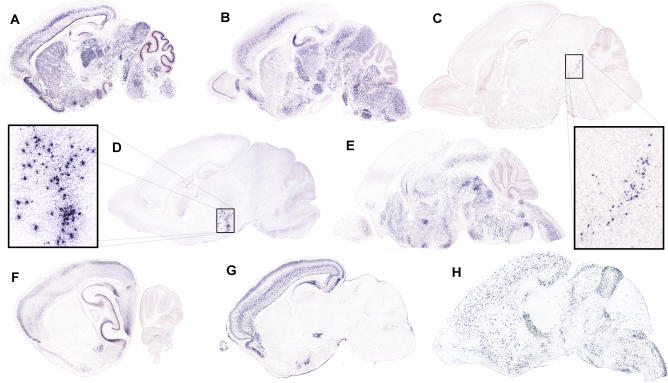
Examples of Gene Expression Patterns Shown are gene expression patterns revealed by nonradioactive robotic ISH on sagittal sections. Digoxigenin-tagged RNA probes hybridized to cellular mRNA are visualized via a serial amplification that produces a blue-purple signal. (A) *Purkinje cell protein 4* in section 4. (B) *Ly6/neurotoxin 1* in section 6. (C) *4931408A02Rik* in section 9 with inset showing localized expression in midbrain neurons. (D) *A230109K2Rik* in section 9 with inset showing localized expression in hypothalamus. (E) *RAS protein-specific guanine nucleotide-releasing factor 1* in section 11. (F) *Gastrin releasing peptide* in section 2. (G) *Nephroblastoma overexpressed gene* in section 4. (H) *Somatostatin* in section 6.

We have assessed the subdivision atlas with a comprehensive test dataset of ~5,000 images of entire sagittal sections from P7 mouse brain produced using robotic ISH for 200 different genes (Table S1). Each gene expression image set spans the left half of the brain and consists of at least 24 sections spaced a maximum of 200 μm apart. Digital images were captured in a bright field microscope at 1.6 μm per pixel resolution. This resolution is sufficient to view individual cell bodies and estimate the strength of expression as reflected by the amount of precipitate in each cell using a previously reported quantification algorithm, Celldetekt [12]. Figure 2 illustrates the types of data—cellular resolution images with diverse expression patterns (insets of Figure 2C and 2D)—that were subjected to annotation by subdivision mesh fittings.

#### Linking expression levels to locations in the atlas.

From the ~24 sections for each gene, we identified the sections that best matched the anatomy represented by standard maps 2, 4, 6, 9, and 11, which collectively are sufficient to characterize all 15 different major anatomical structures in the atlas. The standard maps were deformed to fit appropriate tissue sections (e.g., Figure 1D–1F). We applied Celldetekt to classify the expression levels for cells in the tissue sections, and associated the local levels of expression with the overlying quadrilaterals in the finely subdivided mesh (e.g., Figure 1F). This created a digital dataset of cellular expression levels at all locations across 1,000 mesh-fitted experimental sections representing 200 different genes.

### Knowledge Discovery Using the P7 Mouse Brain Gene Expression Patterns

#### Homologous pattern query.

**Figure 3:**
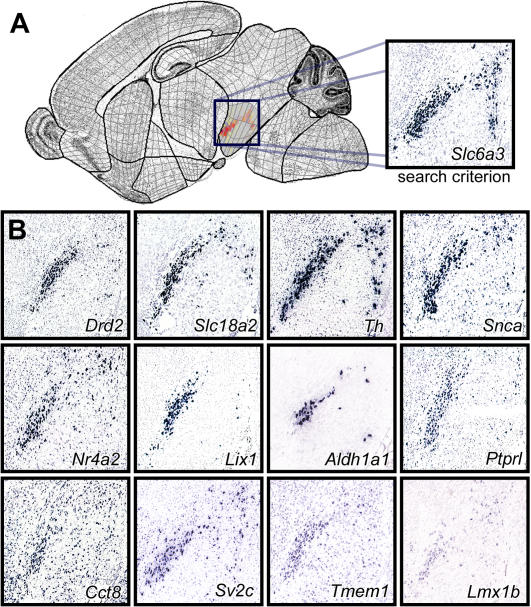
A Search for Genes that Are Expressed in the Substantia Nigra (A) The pattern of Slc6a3 gene expression in and around the substantia nigra at standard section number 6 is set as the query pattern for a search of all 200 expression patterns in the current dataset. Note the color-coded shading of the query pattern, with red indicating the strong expression of *Slc6a3* in the substantia nigra, and grey indicating no expression in the tissue surrounding the substantia nigra. (B) The expression patterns in the substantia nigra pars compacta of the 12 genes found to match the search criterion best are shown: *dopamine receptor 2 (Drd2); vesicular monoamine transporter 2 (Slc18a2); tyrosine hydroxylase (Th); alpha synuclein (Snca);* a gene encoding a nuclear orphan receptor *(Nr4a2); limb expression 1 homolog (Lix1);* a gene encoding an aldehyde dehydrogenase *(Aldh1a1); protein tyrosine phosphatase, receptor type L (Ptprl); chaperonin subunit 8 (Cct8); synaptic vesicle glycoprotein 2c (Sv2c); transmembrane protein 1 (Tmem1);* and *LIM homeobox transcription factor 1 beta (Lmx1b).*

Within the context of an anatomical atlas, comparison of expression patterns in a region of interest provides a mechanism for identifying candidate genes involved in regionalized biological or pathological processes. Idiopathic Parkinson disease (IPD) is a progressive neurodegenerative disorder characterized in part by the loss of dopaminergic neurons in the substantia nigra, resulting in decreased dopamine release in the striatum and severe impairment of motor function. To search for genes potentially involved in IPD, we performed a homologous pattern query for genes in the dataset that best match the expression pattern of *dopamine transporter 1 (Slc6a3),* a marker for dopaminergic neurons, in the substantia nigra (Figure 3A). Genes in the dataset were ranked by their similarity to this query pattern, calculated as the weighted sum of differences in detected cellular expression strengths across all selected quadrilaterals. The top 12 ranked genes are shown in Figure 3B. Query patterns are not limited to genes already in the dataset; as shown in the next example, queries can be performed using user-created query patterns.

#### Expression difference detection.

ISH can reveal changes in gene expression that result from experimental or genetic modification. The present dataset offers the opportunity to obtain a list of genes expressed in a structure that is presumed altered because of such modification. *barrelless (brl)* was chosen to demonstrate this type of analysis. This mutant lacks “barrels,” the discrete cylindrical structures in layer IV of the primary somatosensory cortex that receive sensory input from facial whiskers [13,14]. The phenotype associated with *brl* results from a loss-of-function mutation in calcium/calmodulin-stimulated *adenylate cyclase 1* [15], a cAMP-synthesizing enzyme.

**Figure 4:**
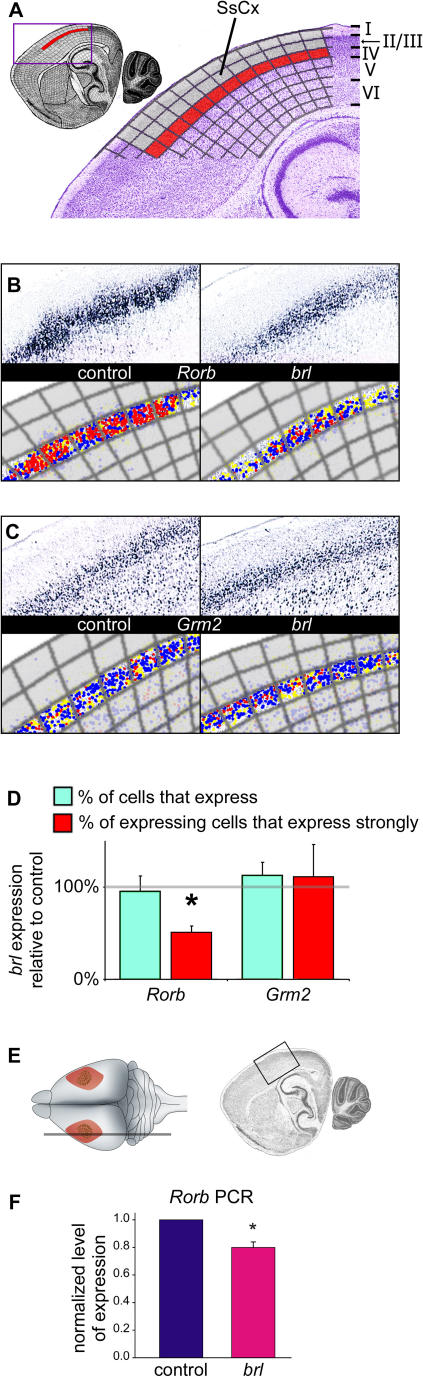
Quantitative Analysis of *Rorb* and *Grm2* Expression in Control and *brl* P7 Brains (A) The dataset of 200 genes was searched using a query pattern defined as strong expression in layer IV of the somatosensory cortex (SsCx) (red) and no expression in layers I and II/III somatosensory cortex (grey) for standard section 2. *Rorb* and *Grm2* were two of the top matches returned. (B) The strong expression *Rorb* in control somatosensory cortex layer IV coincides with the anatomical shape of the barrels that are absent in the *brl* mouse. For both genotypes, cellular expression was detected and color-coded by signal strength using the Celldetekt software, followed by fitting of the appropriate subdivision mesh to the shape of the cortex. A row of 12 quadrilaterals in the subdivision mesh defines the area of comparison in the somatosensory cortex layer IV. Note the greater prevalence of strongly expressing cells (red) in the control tissue. Moderately expressing cells and weakly expressing cells are indicated by blue and yellow, respectively. (C) Quantification of *Grm2* expression in somatosensory cortex layer IV as described for *Rorb* showed no difference in expression strength distribution between control and *brl*. (D) Statistical comparisons between control and *brl* revealed no significant changes in the percentage of somatosensory cortex layer IV cells expressing either *Rorb* (*p* = 0.8) or *Grm2* (*p* = 0.5). However, a significant decrease in the percentage of strongly expressing cells was found for *Rorb* in *brl* (*p* = 0.02), but not for *Grm2* (*p* = 0.8). (E) The somatosensory cortex containing the barrel region was dissected as indicated (highlighted and boxed) and used for quantitative real-time PCR analysis. (F) Consistent with the ISH data, a statistically significant decrease in *Rorb* expression was found in *brl* by quantitative real-time PCR (*p* = 0.008).

A search of our dataset for genes expressed more strongly in layer IV of the barrel field than in layers I and II/III (Figure 4A) returned the transcription factor *retinoid-related orphan receptor beta (Rorb)* (Figure 4B) and the *metabotropic glutamate receptor type 2 (Grm2)* (Figure 4C). We then sought to determine whether there were significant changes in the strength of *Rorb* and *Grm2* expression in *brl* mice. Three pairs of P7 brains from *brl* mutants and their heterozygous littermate controls (possessing intact barrel maps) were subjected simultaneously to robotic ISH using *Rorb* and *Grm2* riboprobes. Cellular expression strengths were determined using Celldetekt [12]. Subdivision mesh atlases were fitted to five adjacent 25-μm-thick tissue sections located between standard sections 2 and 3, and the identical 12 quadrilaterals were selected in each mesh to define a common region of comparison in the barrel field. Although the percentage of cells expressing *Rorb* was similar in control and *brl* tissue (*brl,* 96% ± 17% of control), we found in the *brl* brains a significant (*p* = 0.02) decrease in the relative percentage of cells expressing *Rorb* strongly (*brl,* 51% ± 7% of control) (Figure 4D). By contrast, an identical analysis of *Grm2* expression did not reveal differences in either total expression (*brl,* 113% ± 14% of control) or strong expression (*brl,* 111% ± 35% of control).

To validate the results of our method for difference detection, we performed quantitative real-time PCR. Mice homozygous for *brl* were paired with their heterozygous littermates for *Rorb* expression analysis of the somatosensory cortex containing the barrel region (Figure 4E). We found that *Rorb* expression in the *brl* mice was consistently and significantly lower (*p* < 0.01) than that in the control mice (*brl,* 79% ± 4% of control) (Figure 4F).

### Resources Available

To facilitate distribution and application of the methods of this project, we have made the atlas, dataset, and demonstration queries publicly available online at http://www.geneatlas.org/. The atlas resources consist of the 11 Nissl-stained standard sagittal section images with the major anatomical regions labeled, and the corresponding 11 standard subdivision mesh maps. We also provide an interactive demonstration that allows visitors to deform a map onto an experimental section (as in Figure 1D–1F). All 1,000 reduced-resolution images produced by Celldetekt for this project are also available at this Web site. In addition, most of the ~5,000 images of raw ISH data are available and viewable at http://www.genepaint.org/. The 1,000 images of gene expression patterns can be queried using a graphical search tool that allows users to duplicate the searches in Figure 3 and Figure 4A, as well as to specify different regions of interest and query patterns for their own customized queries.

## Discussion

In this study, we have constructed and applied a subdivision mesh-based atlas to sagittal mouse brain sections revealing the localization of transcripts visualized by ISH. Expression patterns revealed with bacterial artificial chromosome vectors [16], radioactive ISH [17], or immunohistochemistry can readily be subjected to subdivision mesh fitting and thus be represented in the atlas shown here. In addition, it may be possible to capture the architecture of fiber tract connectivity [10], micro MRI data [18], and “tissue voxel-based” microarray-based expression profiles [19] in our subdivision maps. Such multimodality will greatly enhance the discovery power of such an atlas. The subdivision mesh-based atlas can also be used to create tables with sites, levels, and patterns of expression and thus can emulate a text-based annotation procedure [20].

Generating unbiased portraits of gene expression patterns and placing these into a common spatial framework greatly facilitates the discovery of biologically important information. In the case of the *brl* mice, we first searched our 200-gene dataset for genes that are expressed in the developing barrel field region (see Figure 4A). The subsequent detection of down-regulation of the transcription factor gene *Rorb* in *brl* cortex (Figure 4D) raises the possibility that activity-controlled signaling, mediated by *adenylate cyclase 1* in cortical map formation, converges on gene transcription. This discovery also establishes that our annotation can both identify cortical-layer-specific marker genes and estimate quantitative differences in the level of gene expression. Differences in expression levels were more dramatic when using the histology-based method, which accurately delineated the region of interest, than when using quantitative real-time PCR on RNA isolated from a block of cortical tissue (Figure 4D–4F).

The ability to align multiple known expression patterns is the strength of the method described here. We exploited this by searching for genes expressed in a pattern similar to that of *Slc6a3,* which encodes a dopamine transporter and is transcribed in the substantia nigra (see Figure 3). Twelve genes were identified with our homologous pattern search. Seven of these have been previously connected to IPD. *alpha synuclein* and a nuclear orphan receptor *(Nr4a2)* are causative genes in some forms of familial IPD [21,22]. *dopamine receptor 2* and *tyrosine hydroxylase* have been implicated in IPD on the basis of polymorphisms [23,24]. *LIM homeobox transcription factor 1 beta* regulates domamineric neurogenesis [25]. Expression of an aldehyde dehydrogenase *(Aldh1a1)* has been shown recently to be decreased markedly in individuals with IPD [26]. One gene, *vesicular monoamine transporter 2*, is similar to the gene used as the query pattern in that both are involved in monoamine transport.

The five other identified genes have not been previously connected to IPD. *synaptic vesicle glycoprotein 2c* regulates synaptic vesicle exocytosis and has a particularly restricted expression pattern in comparison to other genes in its family, suggesting a potential relationship to the substantia nigra and IPD [27]. The product of *chaperonin subunit 8* is involved in protein folding and assembly [28]. This biochemical property may be a link to IPD because one aspect of this disorder is protein aggregation, mostly of *alpha synuclein* in Lewy bodies. *protein tyrosine phosphatase, receptor type L* encodes a transmembrane receptor with tyrosine phosphatase activity that has been implicated in cell–cell contact [29]. *limb expression 1 homolog* and *transmembrane protein 1* are genes with completely unknown functions. *limb expression 1 homolog* is initially expressed in the precursor cells of the substantia nigra and later in its pars compacta [30]. These results suggest that it is worth considering *synaptic vesicle glycoprotein 2c, chaperonin subunit 8, protein tyrosine phosphatase, receptor type L, transmembrane protein 1,* and *limb expression 1 homolog* as candidates for further investigation into their relationship with IPD. This prototype dataset demonstrates the usefulness of this approach even with a dataset of only 200 genes. By extending this dataset to thousands of genes, our approach would yield a more comprehensive set of candidate genes involved in brain functions and disease mechanisms.

Although the atlas can reliably detect expression in substructures such as the substantia nigra (see Figure 3), cortical layers (see Figure 4A and 4B), and the DG (see Figure 1G), there are limitations in how small a structure the subdivision mesh can consistently locate. This can be addressed by increasing the complexity of the mesh through additional control points. The disadvantage of increased complexity is that fitting the mesh to experimental sections will become more time-consuming. This can be alleviated by focusing on specific anatomical substructures (e.g., just the thalamus), for which new specialized maps could be created.

One of the greatest strengths of the subdivision-based atlas is the ability to fit the maps efficiently and accurately to tissue sections, despite the varying section-to-section deformations introduced by tissue fixation, sectioning, and transfer of sections to slides. By applying this mesh-fitting process, an individual can easily map the expression patterns of 10–20 genes per day. For application of the method to the entire transcriptome, future development efforts should focus on reducing the time involved in the mesh fitting process, e.g., automated fitting based on associating anatomical landmarks with each mesh vertex [31]. In addition, the subdivision method can be extended to create a 3D volumetric subdivision atlas. When coupled with a robust method to stack tissue sections into a 3D volume of gene expression patterns, a 3D subdivision atlas may allow more efficient alignments of expression patterns than a set of two-dimensional maps.

## Materials and Methods

### Non-radioactive ISH.

Tissue preparation, riboprobe preparation, automated ISH, and digitization were performed as previously described [9,32–34] and as described online at http://www.genepaint.org/RNA.htm. Briefly, brains were embedded in OCT and fresh frozen in a chamber that allows stereotaxic alignment of the specimen. Serial sagittal sections at 25 μm thickness were cut with a cryostat through the left half of the brain to just past the midline. Sections from a single specimen were alternately distributed into eight different sets, resulting in a spacing of 200 μm between sections within a set. Each set consisted of approximately 24 sections (four per slide, six slides). Slides were assembled into a flow-through hybridization chamber and placed into position in a Tecan (Mannedorf, Switzerland) Genesis liquid-handling robot, which performs ISH on 192 slides in less than 24 h. Digoxigenin-tagged riboprobes were produced by in vitro transcription from PCR-generated DNA templates using bacteriophage RNA polymerases. Probes were detected by a dual amplification procedure [35].

### Microscopy.

After ISH, slides were cover-slipped and digitally scanned at 1.6 μm/pixel using a custom-made automated Leica (Wetzlar, Germany) microscope [9]. Images were cropped and stored in TIFF format with LWZ lossless compression.

### Atlas creation.

Each standard cross-section was modeled using a Catmull-Clark subdivision mesh [36] partitioned by a network of crease curves. Our subdivision method [37] consisted of two simple transformations: bilinear subdivision that splits each quadrilateral into four subquadrilaterals followed by centroid averaging to reposition vertices (Figure S2). Each quadrilateral in the coarsest mesh was associated with the appropriate anatomical structure. This association is maintained during subdivision.

### Atlas fitting.

ISH sections most similar to the selected maps were visually selected. This was a rapid step requiring less than 1 min for each gene. Standard atlas meshes were then deformed to fit ISH sections using a semi-automated process of computing an affine fit using principal component analysis, performing a local fit using iterated least squares, and verifying visually [11]. Because of the intuitive flexibility of the subdivision meshes, any necessary manual corrections of the mesh fitting were simple and could be performed in 2–5 min per ISH section.

### Pattern query scoring.

As part of an expression pattern similarity query, a total difference score in relation to the query pattern is calculated for each pattern in the dataset. This score, *S,* is the sum of the individual differences, *d,* for each quadrilateral pair within the region of the search, *j:* 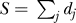 . Each *d* is calculated as a weighted *L*_1_ norm between the vector of the number of cells at different Celldetekt-calculated expression strength levels, *c* = [strong, moderate, weak, none] for the query pattern quadrilateral, *q,* and the current dataset pattern quadrilateral, *p*. Specifically, 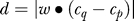 , with weights *w =* [9, 4, 1, 0].

### *Rorb* and *Grm2* analysis.

Each *brl* and littermate control mouse brain pair was subjected to ISH simultaneously. Prior to Celldetekt analysis, image intensity level adjustment was performed on pairs so that the percentage of strongly expressing cells was approximately equivalent from pair to pair. All *p*-values were calculated using two-tailed paired *t*-tests that compared *brl* brain section sets in relation to their control pairs.

### RNA extraction and cDNA generation.

The somatosensory cortex was isolated from *brl* mice (*n* = 16) and heterozygous littermate control mice (*n* = 22) in a total of six group pairs as previously described [38]. Total RNA was extracted, cleaned with DNase I, and then reverse transcribed. Conventional PCR for *Rorb* was performed in samples from heterozygous control and homozygous *brl* animals. The PCR amplicons were sequenced to confirm their identity across control and *brl* samples. The resulting sequences were used for the design of TaqMan (Roche Molecular Systems, Alameda, California, United States) primers and probes for quantitative real-time PCR.

### Quantitative real-time PCR.

The TaqMan probe and primer pair for *Rorb* were as follows: probe, 5′-FAM TCAGAAGAACCACCTGGATGATGAGACCC TAMRA-3′; forward primer, GATTTATTTTGCACTGCAACATGTG; and reverse primer, ACTGCCGTGATAGTTGGTATCTTG. Relative quantification of *Rorb* expression was performed with 18S rRNA as an endogenous control. Each sample was run in triplicate to reduce pipetting error and increase consistency of the results. PCR was carried out at 50 °C for 2 min, 95 °C for 10 min, followed by 40 cycles of 95 °C for 15 s and 60 °C for 1 min. The expected size of the PCR products was confirmed by gel electrophoresis. In addition, a conventional PCR omitting the hybridization probe was run on a thermocycler to verify PCR specificity. Equal amplification efficiency of *Rorb* to 18S rRNA was achieved, validating the relative quantification.

### Animals.

C57BL/6 wild type mice from Jackson Laboratory (Bar Harbor, Maine, United States) were the source of the line of mice used for all 200 genes. The discovery of *brl* mice resulted from a spontaneous mutation in a line from ICR stock at Université de Lausanne [39]. *brl* mice used in our experiments were from the eighth backcross generation of the incipient C57BL/6J-*brl* congenic inbred strain. Genotypes were determined by genomic PCR as described [38]. Data analysis was performed blind to genotype. All animals were treated in compliance with the guidelines of both the U.S. Department of Health and Human Services and Baylor College of Medicine's Animal Care and Use Committee.

## Supporting Information

Figure S1 The Subdivision-Based Anatomical Atlas of the Postnatal Mouse Brain Eleven sagittal maps compose this subdivision-based postnatal mouse brain atlas. The 15 major anatomical structures are color-coded as indicated.
(7.6 MB TIF)

Figure S2 Subdivision Mesh (A) An initial coarse mesh (left). The two transformations of subdivision: bilinear subdivision (middle) and then centroid averaging (right).
(B) The mesh subdivided twice (left), thrice (middle), and four times (right).
(453 KB TIF)

Table S1 List of 200 Riboprobes Used (242 KB DOC)

### Accession Numbers

The Entrez (http://www.ncbi.nlm.nih.gov/gquery/) accession numbers for the genes discussed in this paper are *4931408A02Rik* (XM_354970), *A230109K2Rik* (AK020723), *Aldh1a1* (NM_013467), *alpha synuclein* (NM_009221), *cannabinoid receptor 1* (NM_007726), *chaperonin subunit 8* (NM_009840), *dopamine receptor 2* (NM_010077), *gastrin releasing peptide* (NM_175012), *Grm2* (M92075), *LIM homeobox transcription factor 1 beta* (NM_010725), *limb expression 1 homolog* (NM_025681), *Ly6/neurotoxin 1* (NM_011838), *nephroblastoma overexpressed gene* (NM_010930), *Nr4a2* (NM_013613), *protein tyrosine phosphatase, receptor type L* (NM_011214), *Purkinje cell protein 4* (NM_008791), *RAS protein-specific guanine nucleotide-releasing factor 1* (NM_011245), *Rorb* (NM_146095), *Slc6a3* (NM_010020), *somatostatin* (NM_009215), *Synaptic vesicle glycoprotein 2c* (XM_127490), *transmembrane protein 1* (XM_125775), *tyrosine hydroxylase* (NM_009377), and *vesicular monoamine transporter 2* (NM_013031).

## Abbreviations

3D: three-dimensional
*brl,*: *barrelless*
DG: dentate gyrus
*Grm2,*: *metabotropic glutamate receptor type 2*
IPD: idiopathic Parkinson disease
ISH: in situ hybridization
P7: postnatal day 7
*Rorb,*: *retinoid-related orphan receptor beta;*
*Slc6a3,*: *dopamine transporter 1*
