# Supplementary material for: A Digital Atlas to Characterize the Mouse Brain Transcriptome
Source: PLoS Comput Biol. 2005 Sep 23;1(4):e41. doi: 10.1371/journal.pcbi.0010041 (PMC1215388; doi:10.1371/journal.pcbi.0010041)
Supplement: Supplementary file 1 [file pcbi.0010041.st001.doc]

| **mRNA Sequence** | **GeneID** | **Symbol** | **Description** |
| --- | --- | --- | --- |
| XM_149659 | 68666 | 1110030H18Rik | RIKEN cDNA 1110030H18 gene |
| NM_026342 | 67726 | 1810073G14Rik | RIKEN cDNA 1810073G14 gene |
| AK012533 | 66578 | 2610039C10Rik | RIKEN cDNA 2610039C10 gene |
| BC019153 | 72611 | 2700038I16Rik | RIKEN cDNA 2700038I16 gene |
| XM_125945 | 216440 | 4632413K17Rik | RIKEN cDNA 4632413K17 gene |
| AF358257 | 70967 | 4931408A02Rik | RIKEN cDNA 4931408A02 gene |
| AK020723 | 77764 | A230109K23Rik | RIKEN cDNA A230109K23 gene |
| AB001735 | 11504 | Adamts1 | a disintegrin-like and metalloprotease (reprolysin type) with thrombospondin type 1 motif, 1 |
| AF053980 | 432530 | Adcy1 | adenylate cyclase 1 |
| XM_156060 | 224129 | Adcy5 | adenylate cyclase 5 |
| NM_013467 | 11668 | Aldh1a1 | aldehyde dehydrogenase family 1, subfamily A1 |
| NM_007489 | 11865 | Arntl | aryl hydrocarbon receptor nuclear translocator-like |
| NM_009732 | 11998 | Avp | arginine vasopressin |
| NM_019517 | 56175 | Bace2 | beta-site APP-cleaving enzyme 2 |
| AF153440 | 68010 | Bambi | BMP and activin membrane-bound inhibitor, homolog (Xenopus laevis) |
| BC034862 | 12064 | Bdnf | brain derived neurotrophic factor |
| NM_007553 | 12156 | Bmp2 | bone morphogenetic protein 2 |
| BC013459 | 12159 | Bmp4 | bone morphogenetic protein 4 |
| NM_009768 | 12215 | Bsg | basigin |
| NM_176930 | 319504 | C130076O07Rik | RIKEN cDNA C130076O07 gene |
| NM_009788 | 12307 | Calb1 | calbindin-28K |
| AF395884 | 12325 | Camk2g | calcium/calmodulin -dependent protein kinase II gamma |
| D32007 | 12395 | Cbfa2t1h | CBFA2T1 identified gene homolog (human) |
| NM_009829 | 12444 | Ccnd2 | cyclin D2 |
| NM_009840 | 12469 | Cct8 | chaperonin subunit 8 (theta) |
| NM_133655 | 12520 | Cd81 | CD 81 antigen |
| NM_0909864 | 12550 | Cdh1 | cadherin 1 |
| NM_019707 | 12554 | Cdh13 | cadherin 13 |
| NM_007664 | 12558 | Cdh2 | cadherin 2 |
| X06340 | 12560 | Cdh3 | cadherin 3 |
| NM_009867 | 12561 | Cdh4 | cadherin 4 |
| NM_009868 | 12562 | Cdh5 | cadherin 5 |
| NM_007666 | 12563 | Cdh6 | cadherin 6 |
| NM_007667 | 12564 | Cdh8 | cadherin 8 |
| NM_007668 | 12568 | Cdk5 | cyclin-dependent kinase 5 |
| NM_009871 | 12569 | Cdk5r | cyclin-dependent kinase 5, regulatory subunit (p35) |
| D12490 | 12647 | Chat | choline acetyltransferase |
| XM_145698 | 244059 | Chd2 | chromodomain helicase DNA binding protein 2 |
| AF264050 | 12671 | Chrm3 | cholinergic receptor, muscarinic 3, cardiac |
| X63473 | 12672 | Chrm4 | cholinergic receptor, muscarinic 4 |
| NM_015730 | 11438 | Chrna4 | cholinergic receptor nicotinic a-polypeptide4 |
| NM_009602 | 11444 | Chrnb2 | cholinergic receptor nicotinic ß-polypeptide 2 (neuronal) |
| AF492840 | 108015 | Chrnb4 | cholinergic receptor, nicotinic, beta polypeptide 4 |
| NM_018763 | 54371 | Chst2 | carbohydrate sulfotransferase 2 |
| AF000998 | 12753 | Clock | circadian locomoter output cycles kaput |
| Y18374 | 12801 | Cnr1 | cannabinoid receptor 1 (brain) |
| XM_124763 | 18488 | Cntn3 | contactin 3 |
| NM_009929 | 12822 | Col18a1 | procollagen, type XVIII, alpha 1 |
| AF406641 | 170788 | Crb1 | crumbs homolog 1 (Drosophila) |
| AF156986 | 12952 | Cry1 | cryptochrome 1 (photolyase-like) |
| NM_009986 | 13047 | Cutl1 | cut-like 1 (Drosophila) |
| L12029 | 20315 | Cxcl12 | stromal cell derived factor 1 isoform beta precursor |
| NM_010516 | 16007 | Cyr61 | cysteine rich protein 61 |
| BC013475 | 28295 | D10Jhu81e | DNA segment Chr 10 Johns Hopkins University 81 expressed |
| NM_010014 | 13131 | Dab1 | disabled homolog 1 (Drosophila) |
| NM_007833 | 13179 | Dcn | decorin |
| AF193608 | 60364 | Donson | downstream neighbor of SON |
| AF171079 | 13488 | Drd1a | dopamine Receptor D1A |
| NM_010077 | 13489 | Drd2 | dopamine receptor 2 |
| NM_007893 | 13560 | E4f1 | E4F transcription factor 1 |
| NM_007909 | 13637 | Efna2 | ephrin A2 |
| NM_013508 | 13713 | Elk3 | ELK3, member of ETS oncogene family |
| NM_007936 | 13838 | Epha4 | Eph receptor A4 |
| NM_010155 | 13875 | Erf | Est2 repressor factor |
| NM_011808 | 23871 | Ets1 | E26 avian leukemia oncogene 1, 5' domain |
| NM_008002 | 14165 | Fgf10 | fibroblast growth factor 10 |
| XM_148330 | 14167 | Fgf12 | fibroblast growth factor 12 |
| NM_010201 | 14169 | Fgf14 | fibroblast growth factor 14 |
| AK017829 | 14170 | Fgf15 | fibroblast growth factor 15 |
| M30643 | 14176 | Fgf5 | fibroblast growth factor 5 |
| NM_008008 | 14178 | Fgf7 | fibroblast growth factor 7 |
| J00370 | 14281 | Fos | FBJ osteosarcoma oncogene |
| NM_008023 | 14240 | Foxb2 | forkhead box B2 |
| U36760 | 15228 | Foxg1 | forkhead box G1 |
| NM_008065 | 14390 | Gabpa | GA repeat binding protein, alpha |
| XM_146539 | 209239 | Gan | giant axonal neuropathy |
| X65128 | 14451 | Gas1 | growth arrest specific 1 |
| NM_010285 | 14601 | Ghrh | growth hormone releasing hormone |
| XM_125507 | 14816 | Grm1 | glutamate receptor, metabotropic 1 |
| M92075 | 24415 | Grm2 | glutamate receptor, metabotropic 2 |
| XM_131877 | 108069 | Grm3 | glutamate receptor, metabotropic 3 |
| NM_175012 | 225642 | Grp | gastrin releasing peptide |
| NM_010351 | 14836 | Gsc | goosecoid |
| AF394596 | 230777 | Hcrtr1 | hypocretin (orexin) receptor 1 |
| XM_131908 | 15234 | Hgf | hepatocyte growth factor |
| AF282980 | 235106 | Hnt | neurotrimin |
| AF169620 | 15468 | Hrmt1l1 | heterogeneous nuclear ribonucleoprotein methyltransferase-like 1 (S. cerevisiae) |
| AF209926 | 57295 | Icmt | isoprenylcysteine carboxyl methyltransferase |
| AK087063 | 15926 | Idh1 | isocitrate dehydrogenase 1 (NADP+), soluble |
| NM_008332 | 15958 | Ifit2 | interferon-induced protein with tetratricopeptide repeats 2 |
| NM_010509 | 15976 | Ifnar2 | interferon (alpha and beta) receptor 2 |
| NM_008338 | 15980 | Ifngr2 | interferon gamma receptor 2 |
| AF165984 | 16371 | Irx1 | Iroquois related homeobox 1 (Drosophila) |
| NM_021459 | 16392 | Isl1 | ISL1 transcription factor, LIM/homeodomain (islet 1) |
| NM_013565 | 16400 | Itga3 | integrin alpha 3 |
| BC028778 | 67374 | Jam2 | junction cell adhesion molecule 2 |
| NM_144945 | 246316 | Lgi2 | leucine-rich repeat LGI family, member 2 |
| NM_144556 | 243914 | Lgi4 | leucine-rich repeat LGI family, member 4 |
| NM_008498 | 16869 | Lhx1 | LIM homeobox protein 1 |
| NM_010710 | 16870 | Lhx2 | LIM homeobox protein 2 |
| NM_010711 | 16871 | Lhx3 | LIM homeobox protein 3 |
| AF135415 | 16872 | Lhx4 | LIM homeobox protein 4 |
| MMU61155 | 16873 | Lhx5 | LIM homeobox protein 5 |
| AB031039 | 16874 | Lhx6 | LIM homeobox protein 6 |
| AF113518 | 16876 | Lhx9 | LIM homeobox protein 9 |
| AF351204 | 66643 | Lix1 | limb expression 1 homolog (chicken) |
| AJ296304 | 109594 | Lmo1 | LIM domain only 1 |
| AK013416 | 16909 | Lmo2 | LIM domain only 2 |
| NM_010725 | 16917 | Lmx1b | LIM homeobox transcription factor 1 beta |
| NM_053178 | 94180 | Lpd | lipidosin |
| NM_008377 | 16206 | Lrig1 | leucine-rich repeats and immunoglobulin-like domains 1 |
| BC029082 | 16987 | Lss | lanosterol synthase |
| NM_011838 | 23936 | Lynx1 | Ly6/neurotoxin 1 |
| AF201662 | 17202 | Mc4r | melanocortin 4 receptor |
| NM_008609 | 17388 | Mmp15 | matrix metalloproteinase 15 |
| NM_011308 | 20185 | Ncor1 | nuclear receptor co-repressor 1 |
| AF290472 | 83431 | Ndel1 | nuclear distribution gene E-like homolog 1 (A. nidulans) |
| NM_008684 | 18007 | Neo1 | neogenin |
| NM_016701 | 18008 | Nes | nestin |
| XM_130329 | 18012 | Neurod1 | neurogenic differentiation 1 |
| NM_009717 | 11922 | Neurod6 | neurogenic differentiation 6 |
| NM_009718 | 11924 | Neurog2 | neurogenin 2 |
| NM_033217 | 18053 | Ngfr | nerve growth factor receptor |
| NM_008700 | 18091 | Nkx2-5 | NK2 transcription factor related, locus 5 |
| NM_008714 | 18128 | Notch1 | Notch gene homolog 1 (Drosophilia) |
| NM_008716 | 18131 | Notch3 | Notch gene homolog 3 (Drosophilia) |
| NM_010930 | 18133 | Nov | nephroblastoma overexpressed gene |
| AF273768 | 109648 | Npy | neuropeptide Y |
| X74134 | 13865 | Nr2f1 | nuclear receptor subfamily 2, group F, member 1 |
| U07635 | 11819 | Nr2f2 | nuclear receptor subfamily 2, group F, member 2 |
| S53744 | 18227 | Nr4a2 | nuclear receptor subfamily 4, group A, member 2 |
| X53257 | 18205 | Ntf3 | neurotrophin 3 |
| M33385 | 18212 | Ntrk2 | neurotrophic tyrosine kinase, receptor, type 2 |
| NM_008746 | 18213 | Ntrk3 | neurotrophic tyrosine kinase, receptor, type 3 |
| Y15522 | 18221 | Nudc | nuclear distribution gene C homolog (Aspergillus) |
| NM_181848 | 71648 | Optn | optineurin |
| BC017609 | 18424 | Otx2 | orthodenticle homolog 2 |
| AB019558 | 50873 | Park2 | parkin |
| X63963 | 18508 | Pax6 | paired box gene 6 |
| NM_021568 | 59093 | Pcbp3 | poly(rC) binding protein 3 |
| NM_008791 | 18546 | Pcp4 | Purkinje cell protein 4 |
| NM_011054 | 18575 | Pde1c | phosphodiesterase 1C |
| NM_011058 | 18595 | Pdgfra | platelet derived growth factor receptor alpha polypeptide |
| XM_131313 | 18619 | Penk1 | preproenkephalin 1 |
| AF022992 | 18626 | Per1 | period homolog 1 (Drosophila) |
| NM_011066 | 18627 | Per2 | period homolog 2 (Drosophila) |
| NM_008826 | 18641 | Pfkl | phosphofructokinase, liver, B-type |
| NM_008887 | 11859 | Phox2a | paired-like homeobox 2a |
| NM_008888 | 18935 | Phox2b | paired-like homeobox 2b |
| NM_018797 | 54712 | Plxnc1 | plexin C1 |
| AK009301 | 80294 | Pofut2 | protein O-fucosyltransferase 2 |
| AK017492 | 18976 | Pomc1 | pro-opiomelanocortin-alpha |
| NM_011214 | 19273 | Ptprl | protein tyrosine phosphatase, receptor type, L |
| L20899 | 19417 | Rasgrf1 | RAS protein-specific guanine nucleotide-releasing factor 1 |
| NM_011261 | 19699 | Reln | reelin |
| XM_129222 | 225998 | Rorb | RAR-related orphan receptor beta |
| X83542 | 20238 | Sca1 | spinocerebellar ataxia 1 homolog (human) |
| NM_031196 | 20509 | Scl19a1 | solute carrier family 19 (sodium/hydrogen exchanger), member 1 |
| X85993 | 20346 | Sema3a | sema domain, immunoglobulin domain (Ig), short basic domain, secreted, (semaphorin) 3A |
| NM_013660 | 20354 | Sema4d | sema domain, immunoglobulin domain (Ig), transmembrane domain (TM) and short cytoplasmic domain, (semaphorin) 4D |
| X97818 | 20357 | Sema5b | sema domain, seven thrombospondin repeats (type 1 and type 1-like), transmembrane domain (TM) and short cytoplasmic domain, (semaphorin) 5B |
| NM_011352 | 20361 | Sema7a | sema domain, immunoglobulin domain (Ig), and GPI membrane anchor, (semaphorin) 7A |
| NM_009250 | 20713 | Serpini1 | serine (or cysteine) proteinase inhibitor, clade I, member 1 |
| X76290 | 20423 | Shh | sonic hedgehog |
| BB273238 | 320038 | Shhrs | sonic hedgehog regulated sequence |
| XM_127012 | 66354 | Skiip | SKI interacting protein |
| XM_140760 | 25549 | Slc18a2 | solute carrier family 18 (vesicular monoamine), member 2 |
| NM_015747 | 20515 | Slc20a1 | solute carrier family 20, member 1 |
| NM_010020 | 13162 | Slc6a3 | solute carrier family 6 (neurotransmitter transporter, dopamine), member 3 |
| AF013604 | 15567 | Slc6a4 | solute carrier family 6 (neurotransmitter transporter, serotonin), member 4 |
| AF144628 | 20563 | Slit2 | slit homolog 2 (Drosophila) |
| AF033261 | 20617 | Snca | synuclein, alpha |
| NM_019973 | 20658 | Son | Son cell proliferation protein |
| XM_128755 | 50850 | Spg4 | spastic paraplegia 4 homolog (human) |
| AF338472 | 259302 | Srgap3 | SLIT-ROBO Rho GTPase activating protein 3 |
| NM_009215 | 20604 | Sst | somatostatin |
| BI736186 | 64051 | Sv2a | synaptic vesicle glycoprotein 2a |
| AI852232 | 64176 | Sv2b | synaptic vesicle glycoprotein 2b |
| AA241127 | 75209 | Sv2c | synaptic vesicle glycoprotein 2c |
| AF117207 | 20974 | Syngr3 | synaptogyrin 3 |
| NM_009311 | 21333 | Tac1 | tachykinin 1 |
| NM_009377 | 21823 | Th | tyrosine hydroxylase |
| NM_009379 | 21832 | Thpo | thrombopoietin |
| NM_009384 | 21844 | Tiam1 | T-cell lymphoma invasion and metastasis 1 |
| NM_011589 | 21853 | Timeless | timeless homolog (Drosophila) |
| NM_011599 | 21885 | Tle1 | transducin-like enhancer of split 1, homolog of Drosophila E(spl) |
| NM_009389 | 21887 | Tle3 | transducin-like enhancer of split 3, homolog of Drosophila E(spl) |
| XM_125775 | 216131 | Tmem1 | transmembrane protein 1 |
| NM_031997 | 83921 | Tmem2 | transmembrane protein 2 |
| NM_011607 | 21923 | Tnc | tenascin C |
| NM_013696 | 22045 | Trhr | thyrotropin releasing hormone receptor |
| AF220039 | 94090 | Trim9 | tripartite motif protein 9 |
| NM_032000 | 83925 | Trps1 | trichorhinophalangeal syndrome I (human) |
| NM_009441 | 22129 | Ttc3 | tetratricopeptide repeat domain |
| NM_011668 | 22215 | Ube3a | ubiquitin protein ligase E3A |
| AB025313 | 22223 | Uchl1 | ubiquitin carboxy-terminal hydrolase L1 |
| NM_021290 | 22226 | Ucn | urocortin |
| NM_013919 | 30941 | Usp21 | ubiquitin specific protease 21 |
| NM_011695 | 22334 | Vdac2 | voltage-dependent anion channel 2 |
| Z47205 | 235320 | Zbtb16 | zinc finger and BTB domain containing 16 |
